# Approach to Pregnancy Affected by Kell Alloimmunization

**DOI:** 10.1155/2024/1929147

**Published:** 2024-07-23

**Authors:** Serdar Aykut, Suleyman Cansun Demir, Ismaıl Cuneyt Evruke, Mete Sucu, Fatma Islek Uzay, Mesut Avan, Ozge Keles Bayer, Emre Yalcin

**Affiliations:** Department of Obstetrics and Gynecology Division of Maternal Fetal Medicine Cukurova University School of Medicine, Adana, Türkiye

## Abstract

Hemolytic disease of the fetus and newborn (HDFN) is the development of anemia, hyperbilirubinemia, and finally hydrops fetalis in the fetus when antibodies to antigens on the surface of erythrocytes are transferred from the placenta to the fetus. The most common cause is D-HDFN. K (KEL1) from the Kell blood group system is the most potent immunogenic antigen after D among all blood group antigens. K-HDFN occurs in 0.1–0.3% of pregnant women. It accounts for 10% of cases of antibody-mediated severe fetal anemia. We present a successful management of Kell alloimmunization in a pregnant woman who had 3 times pregnancy loss with hydrops fetalis due to K-HDFN and who was proven to have K-HDFN in the postnatal period in her last pregnancy.

## 1. Introduction

Hemolytic disease of the fetus and newborn (HDFN) is still a serious complication of pregnancy. This is caused by maternal specific IgG autoantibodies passing through the placenta binding to erythrocytes and causing progressive fetal hemolysis. More than 300 blood group antigens have been identified in the “Terminology of Blood Groups” reports published by the International Society of Blood Transfusion (ISBT) in 2004 and 2007 [1, 2]. More than 50 of these antigens have been reported to cause HDFN [3]. Among these, anti-D immune globulin is the most common antibody causing HDFN requiring IUT [1, 2]. K (KEL1) from the Kell blood group system is the most potent immunogenic antigen after D among all erythrocyte surface antigens [4]. A successful management of Kell alloimmunization in a pregnant woman who had 3 times pregnancy loss with hydrops fetalis due to K-HDFN and who was proven to have K-HDFN in the postnatal period in her last pregnancy is presented here.

## 2. Case

A 31-year-old patient with gravida 7, parity 6, and 3 living children presented to our perinatology clinic at 27 weeks of gestation.

The patient delivered her first pregnancy in 2012 and her second pregnancy in 2015 by spontaneous vaginal delivery. The two children born were alive and healthy.

In 2018, she was admitted to the hospital with her 3rd pregnancy at 29 weeks due to hydrops fetalis, polyhydramnios, and early onset of labor pains. The baby died 1 day after birth due to preterm labor and existing anomalies.

Again in 2018, she delivered her 4th pregnancy at 24 weeks by normal vaginal delivery with the diagnoses of fetal hydrops fetalis and intrauterine dead fetus.

In 2020, she delivered her 5th pregnancy at 28 weeks by normal vaginal delivery due to hydrops fetalis and preterm labor. The baby died 1 day after birth.

Our patient, who carried out her pregnancies so far outside the city, was told that there was no problem in the genetic examinations performed on our patient and the fetuses, but no records could be found.

In 2021, she came to our clinic at 28 weeks of her 6th pregnancy, this time from out of the city. In the examinations performed, it was determined that her blood group was B+, antibody screening was positive, and titer was reported as 1/8. Hydrops fetalis and polyhydramnios were detected in the fetus. The pregnant woman who underwent emergency cesarean section due to preterm delivery was found to have K antigen in postnatal routine laboratory examinations. She was hospitalized in the neonatal intensive care unit for a long time and discharged in good health after the necessary medical care and treatments were provided.

In 2023, our patient, who had her 7th and last pregnancy, presented to our perinatology clinic for the first time at 27 weeks. Routine investigations were unremarkable. Indirect Coombs test was reported as positive. Antibody screening was compatible with anti-K and titer was reported as 1/4. Sonographic examination revealed abdominal ascites, pericardial effusion, scalp edema, and polyhydramnios. Fetal anemia was evaluated by MCA Doppler and PSV (peak systolic velocity) was found to be 54 cm/sec, above 1.5 MoM.

Since the findings supported fetal anemia due to K-HDFN, antenatal steroid administration and neuroprotective MgSO_4_ administration were performed and intrauterine transfusion preparations were started.

We prepared an erythrocyte suspension with a final hematocrit value of 80%, taken within the last 5 days for transfusion. The characteristics of the prepared erythrocyte suspension were blood group 0‐, especially Kell antigen negative, and CMV negative, and 25 Gy-irradiated fresh erythrocytes were used. Amount of erythrocytes to be transfused (ml) = fetoplacental unit volume (ml) × (target − initial hematocrit). Fetoplacental unit volume = 1.046 + estimated fetal weight (g) × 0.14 [5].

Intrauterine transfusion was started transplacentally with a 20-gauge spinal needle inserted transplacentally through the umbilical cord insertion into the umbilical vein. Blood samples were collected for fetal blood group, hemoglobin, and hematocrit. Fetal blood group antigen was reported as O-, hemoglobin was 5.5 g/dl, and Htc (hematocrit) was 18.3%. As a result of the calculations, 40 cc of prepared erythrocytes was given. Control blood count was taken and the procedure was terminated. Htc was 32%. The patient was not discharged because she was from out of the city. The 2nd transfusion was decided to be performed 10 days after the first transfusion since the target hematocrit values could not be reached and the same clinical findings persisted. 40 cc of erythrocyte suspension with an Htc of 80% was transfused. Initial hemoglobin value was 3.9 g/dl and Htc was 11.9%. Control hemoglobin value was 8.2 g/dl and Htc was 24.4%.

Sonographic examination performed after transfusion revealed that the fetus was 30 weeks and approximately 1560 g, consistent with the gestational week.

Since there was no improvement in the clinical condition and the blood given after the first transfusion was destroyed in a short time, it was decided that it would be more appropriate to perform the third transfusion after delivery in terms of neonatal mortality and morbidity after consultation with the neonatology clinic, and a live male baby weighing 1500 g, with an APGAR 3 at 1 minute and APGAR 7 at 5 minutes, was delivered by cesarean section.

The baby was taken to the neonatal intensive care unit and his blood gas pH was 7.26 and PaO_2_ was 85%. He was intubated and followed up because of difficulty in breathing. Exchange transfusion was performed 1 time. Necessary medical treatment and care were provided in the intensive care unit for approximately 28 days. At the end of the 28th day, he was transferred to the neonatal service. She was discharged 1 day later in good condition.

## 3. Discussion

Hemolytic disease of the fetus and newborn is the development of anemia, hyperbilirubinemia, and finally hydrops fetalis in the fetus when antibodies to antigens on the surface of erythrocytes are transferred from the placenta to the fetus. The severity of the disease depends on the depth of anemia. After the widespread antenatal and postnatal use of anti-D ımmunoglobulin, maternal alloimmunization with other erythrocyte antigens has been increasing [1, 2]. K-HDFN occurs in 0.1–0.3% of pregnant women. It accounts for 10% of cases of antibody-mediated severe fetal anemia [6]. Kell blood group system antigen is expressed in erythroid and myeloid lineage cells and in small amounts in many organs, including lymphoid organs, muscle (skeletal and cardiac muscles), and the nervous system [7]. These antigens begin to appear on the surface of fetal erythrocytes at 12 weeks of gestation. Clinical signs of K-HDFN can be detected as early as 18 weeks of gestation in the intrauterine period and can cause rapidly developing severe fetal anemia [8]. The Kell blood group system antigen consists of 23 different members. The most common of these are Kell (K or K1) and Cellano (k or K2). Approximately 92% of the population is Kell antigen negative. Most individuals who are Kell antigen positive are heterozygous. Unlike anemia caused by other antibodies, anemia in K-HDFN is caused not only by hemolysis but also by suppression of erythropoiesis as erythroid precursor cells are affected [9]. The management of K-HDFN is similar to that of D-HDFN. A fetus affected in a previous pregnancy should be closely monitored following conception. The low sociocultural level of our patient and the fact that she did not receive adequate and effective health care services may be explained by the fact that the cause of the fetuses diagnosed with hydrops fetalis in her 3rd, 4th, and 5th pregnancies was not revealed. In her 6th pregnancy, she came to our clinic from out of the city with a diagnosis of hydrops fetalis at 28 weeks of gestation, and unfortunately, the etiology of hydrops fetalis was left to the end of delivery because she was in labor pains when she arrived, and in the examinations performed, hydrops fetalis due to K-HDFN was diagnosed.

K-HDFN should be considered in the differential diagnosis of hydrops fetalis and pregnancy loss, especially in cases with anemia in the newborn. The fact that our patient had a history of hydrops fetalis pregnancy loss and anemia due to K-HDFN in her last delivery helped us to diagnose hydrops fetalis due to K-HDFN. In D alloimmunization, the titer limit of the indirect Coombs test that may cause fetal anemia is considered to be 1/32 [3]. A study assessed 156 K-positive pregnancies (i.e., mothers with anti-K alloantibodies) and found that a critical titer of 32 was 100% sensitive for identifying fetuses affected by anti-K alloimmunization [10]. In another study, 17 pregnancies affected by hemolytic disease of the newborn and fetus were identified. All but 1 patient had serum titers of 32 or higher. The only exception was a pregnant patient with an anti-K titer of 8 who developed fetal hydrops. The study suggested that a critical titer of 1 : 8 may be more sensitive [11]. A 2019 study by Vlachodimtropoulou et al. found that an anti-K titer of 32 predicted the need for intervention with 100% sensitivity, consistent with the results obtained by Vlachodimtropoulou et al. [12]. One of the largest retrospective studies analyzed more than 1,000 anti-K-immunized pregnancies in the Netherlands. The study reported that a critical titer of 4 had a sensitivity of 100% for detecting fetuses requiring intervention, but specificity at this titer level was only 26%. They also reported that a titer of 16 may be appropriate as sensitivity is still high (96%) and specificity has increased significantly (66%) [13]. Additional studies have shown that the anti-K titer in affected fetuses is still controversial. Completely eliminating anti-K titers from the maternal alloimmunization testing algorithm, as suggested by ACOG Practice Bulletin 192, increases the number of unaffected fetuses being monitored by techniques that reflex immediately to invasive testing and treatment [14].

High sensitivity is a crucial component of screening tests, particularly for potentially fatal conditions. An anti-K titer threshold of 8 using a standard tube testing method would impart a lower risk of missing a fetus requiring intervention (1.7%) than the contemporary risk of fetal adverse events incurred by IUTs as reported by a world-renowned referral center for fetal therapy (3.3%) [15]. Interpretation of titers should also include parallel titrations to compare titer results of a current specimen with the previous specimen, as a change of 2 dilutions or greater is also suggestive of increased risk [16]. Anti-K is acknowledged to be a unique alloantibody capable of mediating both marked erythropoietic suppression and immune-mediated hemolysis. The statements in ACOG Practice Bulletin 192 regarding the futility of anti-K titers are not supported by the preponderance of published studies in the literature, nor are they supported by the specific studies cited in the bulletin. Anti-K titers represent a helpful early test when assessing the risk of HDF in patients with a history of alloimmunization and would likely decrease the number of fetuses subjected to invasive testing due to false positive MCA results [17].

No correlation has been found between maternal antibody titers and the severity of HDFN. Severe disease can be seen at low titers. In the follow-up of the next pregnancy, fetal anemia should be monitored with MCA Doppler at two-week intervals starting from the 18th gestational week. Many studies have shown that MCA Doppler is the best noninvasive method to predict anemia. In the past, an invasive method based on spectrophotometric measurement of bilirubin in the amniotic fluid was used to predict anemia [18]. Amniocentesis is no longer a routine method for the detection of fetal anemia because MCA measurement is more sensitive and specific in detecting anemia [19].

Once anemia is diagnosed, it is treated with intrauterine transfusion. The success rate of intrauterine transfusion varies according to the experience of the center and the presence of signs of hydrops fetalis but averages around 90%. Intrauterine treatment of HDFN has significantly reduced perinatal morbidity and mortality [20].

## 4. Conclusion

K-HDFN in fetuses with hydrops fetalis is rarely encountered in obstetric daily practice. It may not be considered in the differential diagnosis of fetuses with hydrops. Pregnant women with fetal loss due to hydrops fetalis or who have received previous blood transfusions are at risk of exposure to non-RH blood group antigens that can cause alloimmunization. Investigation of erythrocyte surface antigens other than RH blood group system antigens in these patients is extremely important in reducing perinatal mortality and morbidity.

## Data Availability

All the data can be requested from the corresponding author upon reasonable request.

## Additional Points

*What Is Already Known about This Topic?* Hemolytic disease of the fetus and newborn is still a serious complication of pregnancy. More than 300 erythrocyte surface antigens have been identified in the “Terminology of Blood Groups” reports published by the International Society of Blood Transfusion. More than 50 of these antigens have been reported to cause hemolytic disease of the fetus and newborn (HDFN). K (KEL1) from the Kell blood group system is the most potent immunogenic antigen after D among all erythrocyte surface antigens. K-HDFN in fetuses with hydrops fetalis is rarely encountered in obstetric daily practice. *What Does This Study Add?* K-HDFN is very rarely encountered in obstetric practice. It may not be considered in the differential diagnosis of fetuses with hydrops. Investigation of erythrocyte minor blood group incompatibilities in pregnant women with hydrops fetalis is extremely important in reducing perinatal mortality and morbidity. This case report draws attention to minor blood group antigen incompatibilities.
